# Using information and communication technologies (ICTs) to solve the repressed demand for primary dental care in the Brazilian Unified Health System due to the COVID-19 pandemic: a randomized controlled study protocol nested with a before-and-after study including economic analysis

**DOI:** 10.1186/s12903-022-02101-9

**Published:** 2022-04-07

**Authors:** Karina Haibara Natal, Thais Gomes Machado, Fabiana Bracco, Luiz Ivan Lemos, Maria Eduarda Vigano, Gabriela Manco Machado, Jhandira Daibelis Yampa-Vargas, Daniela Prócida Raggio, Fausto Medeiros Mendes, José Carlos Pettorossi Imparato, Edson Hilan Gomes Lucena, Yuri Wanderley Cavalcanti, Cícero Inacio Silva, Guido Lemos Souza Filho, Mary Caroline Skelton Macedo, Fernanda Campos Almeida Carrer, Mariana Minatel Braga

**Affiliations:** 1 Department of Pediatric Dentistry, School of Dentistry, University of São Paulo, Lineu Prestes Avenue, 2227, São Paulo, SP 05508000 Brazil; 2 Department of Clinical and Social Dentistry, Federal University of the Paraiba, Paraiba, Brazil; 3 Federal University of São Paulo, São Paulo, Brazil; 4 Department of Computer Science, Federal University of the Paraiba, Paraiba, Brazil; 5 Department of Restorative Dentistry, School of Dentistry, University of São Paulo, São Paulo, Brazil; 6 Department of Social Dentistry, School of Dentistry, University of São Paulo, São Paulo, Brazil

**Keywords:** Child, Teledentistry, Telemonitoring, Teleorientation, Coronavirus infections

## Abstract

**Background:**

With the COVID-19 pandemic, thousands of children had their dental care interrupted or postponed, generating a pent-up demand for primary care. To minimize the impact of this outage, information and communication technologies (ICT) could be an alternative. The aim of this study is to elucidate the impact of implementing the ICTs in primary dental care for children on resolving the pent-up demand for primary dental care to children in the national health system service (SUS) due to the COVID-19 pandemic.

**Methods:**

Different research strategies are being proposed to demonstrate such effect and extrapolating findings to a real-world context to guide further research, practice and policies: two clinical trials (one randomized controlled by the waiting list trial (RCT) and a before-and-after study), one simulation study to prospect trial results to a broader population and three economic evaluations using different effects. Children enrolled in a reference dental unit will be invited to participate in the before-and-after study for trials. The first 368 families will be randomized for the RCT to the intervention vs waiting list. All participants will receive the intervention, but the waiting list group will be assessed before the intervention is available for them. The intervention comprises standardized non-face-to-face primary dental care using the V4H platform. The problem-solving and the family's perception will be the primary outcomes set for the before-and-after study and RCT, respectively. They will be measured 2 weeks after randomization. Based on trial findings, we will develop theoretical models to estimate how the intervention could benefit the population included in the national health system.  Three economic evaluations will be carried out considering different trial effects (cost-effectiveness analyses). A societal perspective and the pandemic time horizon will be considered. Possible social impact (inequalities) will also be explored.

**Discussion:**

This ongoing trial may be an essential contribution to clarify positive and negative aspects related to the use of technologies for non-face-to-face dental care for children. Trial products may bring relevant contributions to the pandemic context and the post-pandemic period. Potential benefits may be feasible to implement and preserve in the health system even in the post-pandemic period.

*Trial registration* Clinicaltrials.gov registration NCT04798599 (registered March 2021).

**Supplementary Information:**

The online version contains supplementary material available at 10.1186/s12903-022-02101-9.

## Background

A drastic repressed demand for primary dental care has been created since the beginning of the COVID-19 pandemic [1–3]. Due to the health professionals' higher risk of contamination [4] associated with aerosol generation [5, 6] dentists have been orientated to postpone elective treatments in different locations [7]. A reduction of almost 90% in primary dental care for children was noted in the Brazilian National Health System (Unified Health System—SUS) compared to the beginning of the pandemic time [8]. This scenario, therefore, contributed to thousands of children lacking primary care, both for their non-operative and operative needs, representing essential pent-up demand for Public Health and should urgently guide policies to deal with this context.

The use of technologies could be a meaningful strategy for minimizing such impact on the health systems. Teledentistry has been demonstrated to be equivalent (or even valid) to face-to-face sessions for different purposes related to primary care, as screening, consultations, orientations and referrals [9, 10]. The use of technology in dental health care has been demonstrated to result in health equity services, reduction in waiting time, and costs with treatment [9]. Its benefit has been noted, especially in school-based programs and patients with limited access to care. The pandemic has created an unexpected and unusual difficulty to access that could be overcome by using this kind of technology.

We hypothesized that using the Teledentistry resources in primary dental care for children might aid to solve some demands that do not necessarily require a face-to-face appointment. Indeed, a teleconsultation could reduce the waiting time for seeing a professional and comfort the patients/parents, solve patients’/parents' doubts, orient about habits and conducts, and refer to other services when necessary. In addition, the pandemic could be a window of opportunity to introduce regular oral health care [11], and the potential for implementation should also be explored.

On the other hand, some barriers have also been associated with this promising alternative, such as infrastructure, patients' motivation/compliance, stakeholders' resistance, and legal and security issues [9, 12]. They are more frequent in developing countries, in which there is a lack of robust evidence about the use of this technological strategy. Indeed, very few assessment studies about ICTs have been performed in developing countries [9]. Understanding which possible adaptations to the intervention are needed to overcome these barriers is crucial to its effective implementation because the core components of the intervention must be retained for effectiveness [13].

Broad implementation of any intervention in diverse practice systems demands understanding the perspectives of different stakeholders who will affect and be affected by such intervention [14]. When planning health actions, users' judgment/satisfaction with health services are also imposing indicators to consider [15]. Besides, the allocation of resources is another relevant aspect for planning actions within health services. It is worth knowing whether the proposed strategy represents an efficient allocation of resources to substitute what has been currently done (or in such case, what is not being done). Thus, economic evaluations can be used as additional tools for making political and economic decisions [16–18].

The present trial aims to elucidate the impact of implementing the ICTs in primary dental care for children on resolving the pent-up demand for primary dental care to children in the SUS due to the COVID-19 pandemic. In this sense, different research strategies are being proposed to demonstrate such effect and extrapolating findings to a real-world context to guide further research, practice and policies. Trial products may bring relevant contributions to the pandemic context and the post-pandemic period.

The following specific objectives are defined:

1. To identify the repressed demand for the outage of dental care and the benefits and difficulties, as well as the perception of users, in the implementation of new strategies of non-face-to-face care based on technology (teleservice), using a primary care cell unit for children and prospecting, through models, this situation for the scenario of the Unified Health System (SUS).
2. Perform different forms of economic evaluation to measure whether the gains achieved with the implementation of teleservice (telescreening, teleorientation and telemonitoring) compensate for the additional costs possibly associated with it, or whether they are associated with a long-term resource savings, considering the implementation  dimensioned for the Brazilian public health system.
3. Explore how the introduction of these technologies could benefit the SUS in the trans and post-pandemic period, contribute to the correction of possible inequities in the health care and other social aspects, as well as result in possible differences when different Brazilian scenarios are explored.

## Methods

This manuscript was written following the Standard Protocol Items: Recommendations for Interventional Trial (SPIRIT) [19] for clinical studies (Additional file 1) and potential items to be included in Health Economics Analysis Plans (HEAPs) for Trial-Based Economic Evaluations [20] for economic evaluation (Additional file 2). The primary trial was registered on Clinicaltrials.gov (TeleDent-COVID19—registration NCT04798599) in 2020.

### Study setting

A mobile dental unit was chosen as the reference unit to collect outcomes related to implementing technologies for primary dental care for children. This unit is in the Greater São Paulo and has been providing dental care for children since 2014 (FAPESP-2012/50716-0). Since then, approximately 10,500 operative and non-operative dental appointments have been accomplished.

**Fig. 1:**
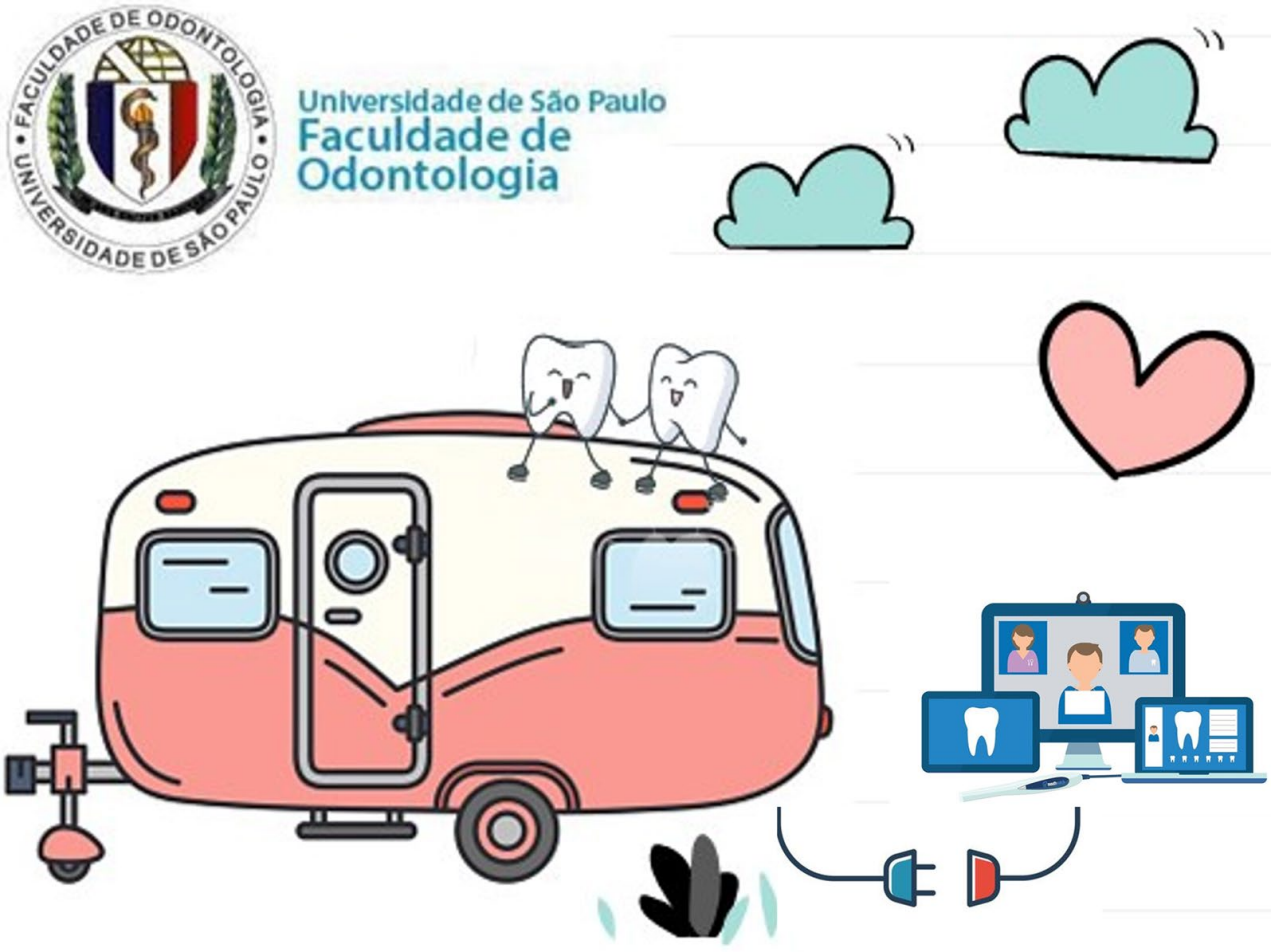
Teletrailer program logo—program for non-face-to-face primary dental care for children, the intervention tested in TeleDent-COVID-19

Currently, 750 children are enrolled for being cared for in this unit. They have participated in some previous clinical trials whose Recruitment was done at the unit. In the pre-pandemic period, nearly 40 children were regularly seen per week. Dental care was part of research protocols developed in the unit and fulfilled patients' needs. Emergency care was also part of the routine. On average, eight emergency visits were performed per month. Toothache and prolonged tooth retention were the main reasons for them. Due to the current pandemic scenario, face-to-face care was suspended, causing our team to seek new alternatives for welcoming these patients. Then, an intervention for non-face-to-face caring using technologies was designed to overcome such situations and the Teletrailer program was launched (Fig. 1).

### Trial design and participant timeline

**Fig. 2:**
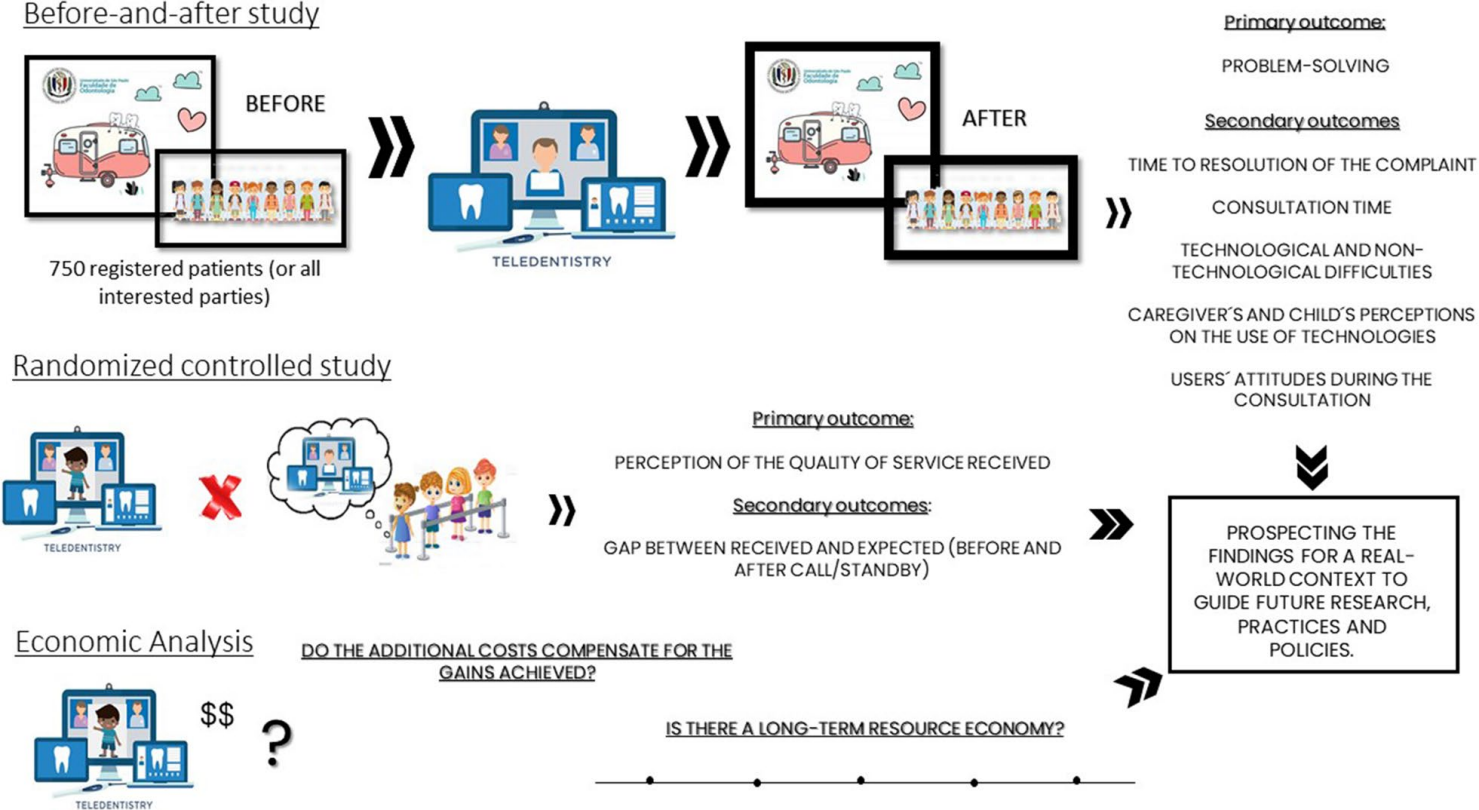
The architecture of studies proposed to demonstrate the effect of implementing the non-face-to-face primary dental care for children using technological resources and extrapolating these findings to a real-world context to guide further research, practice and policies

A randomized controlled trial (RCT) was designed to be nested in a before-and-after study to cover different outcomes explored when implementing the ICTs in primary dental care for children (Fig. 2). A health system-simulated scenario was set using a mobile dental unit as a cell unit for primary data collection.

The primary outcome was set in the RCT. Such a study was controlled by participants' waiting list and measures users' /parents' perceptions about the health care offered and permitted using the participants waiting for dental care as reference (Fig. 2). The before-and-after study collected data related to the natural implementation of ICT in the Teletrailer program.

**Fig. 3:**
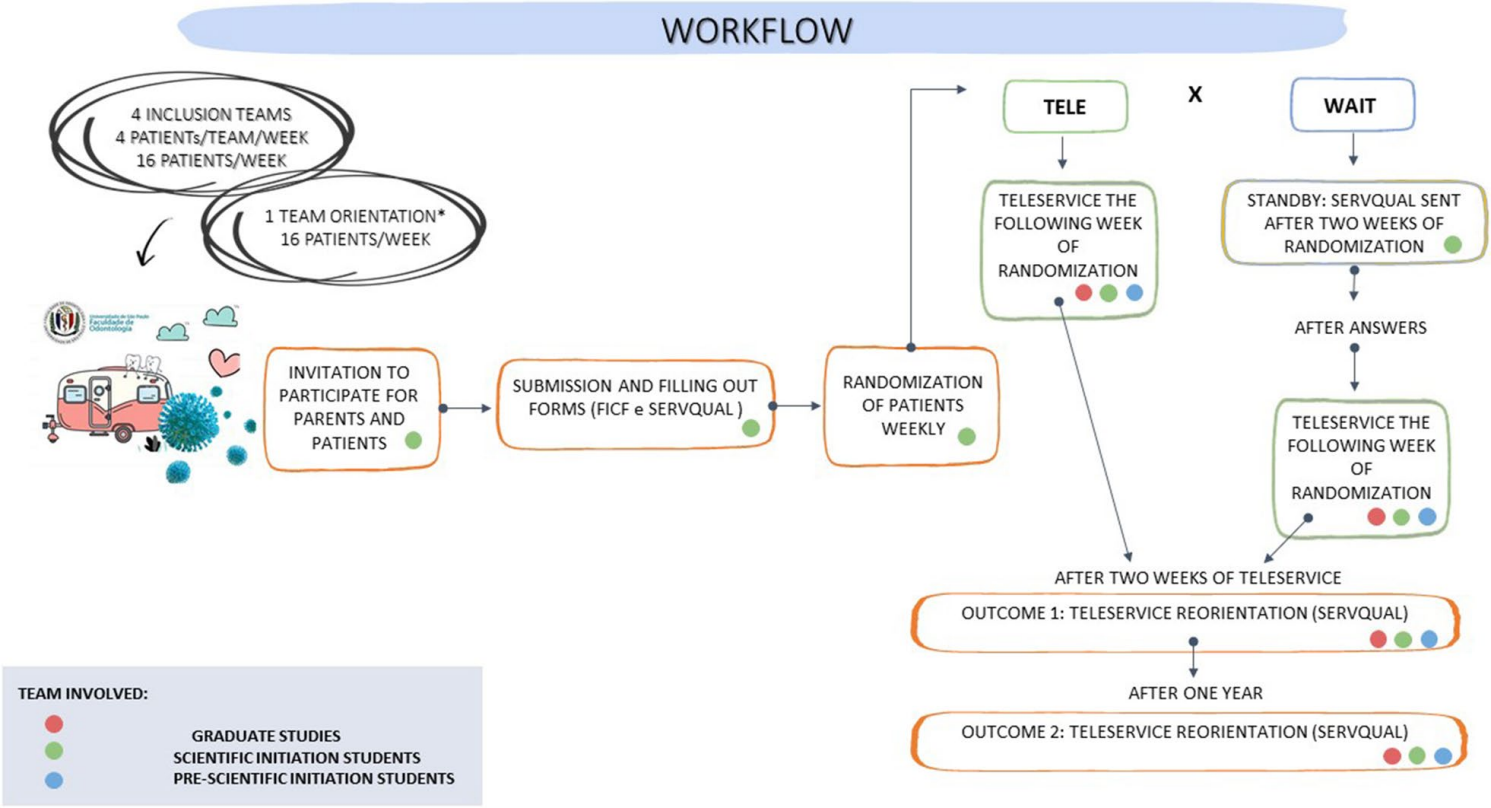
Schematic diagram representing the workflow for conciliating the studies **and** the time schedule of enrolment, interventions, assessments, and visits for participants

The situation before the non-face-to-face intervention was considered the control to the before-and-after study. To measure it, besides the consultation, researchers will establish telephone contact with all registered patients and collect information about pain during the trans pandemic period, search for dental care in other public or private units and other possible complaints that occurred during this period. Besides, information reported during the teleconsultation will be considered as that. Thus, we will have an overview of the impact of the outage of care in the unit after social distancing. Outcomes are related to pen-up demand solving, as well as the strengths and barriers of the program implementation. They were collected in the "after" moment, two weeks after implementing the intervention. Other outcomes in this study design were collected after one year from intervention as detailed further (Figs. 2, 3).

Included children will continue to be followed every six months, following the systematic proposal when implementing technology-mediated interventions until the return of face-to-face activities is possible and the entire structure of the dental unit is prepared for dental care. The systematic recording of new complaints and complications will be maintained both during the consultation and in spontaneous contacts of the participants.

Apart from these clinical studies, simulation models will be used to prospect the ideally found in the cell unit to a SUS-simulated scenario, predicting the actual implementation on regular health units in SUS. Economic and social impact (including inequalities exploration) will also be explored in secondary analyses exploring data collected in the mentioned primary studies (Fig. 2). The following sessions will be described considering each of the research strategies mentioned, as illustrated in Fig. 2. The individual items related to the study design were described separately for each one. For the clinical studies, some sessions were merged to a broader comprehensive.

### The before-and-after study

#### Main purpose

Measuring the natural impact on pent-up demand solving and other effects resulting from telecare implementation in primary care for children.

#### Participants: eligibility criteria

All children enrolled for dental care in the unit were eligible for this trial. Their parents/caregivers will be contacted and invited to be part of the non-face-to-face program. After several attempts, children who cannot be contacted will be excluded from the sample. Children or guardians who do not agree to participate in the research will be computed as an outcome, as described further. In this case, teleconsultation may be performed if they desire, but without other data collection for research purposes.

#### Interventions

**Fig. 4:**
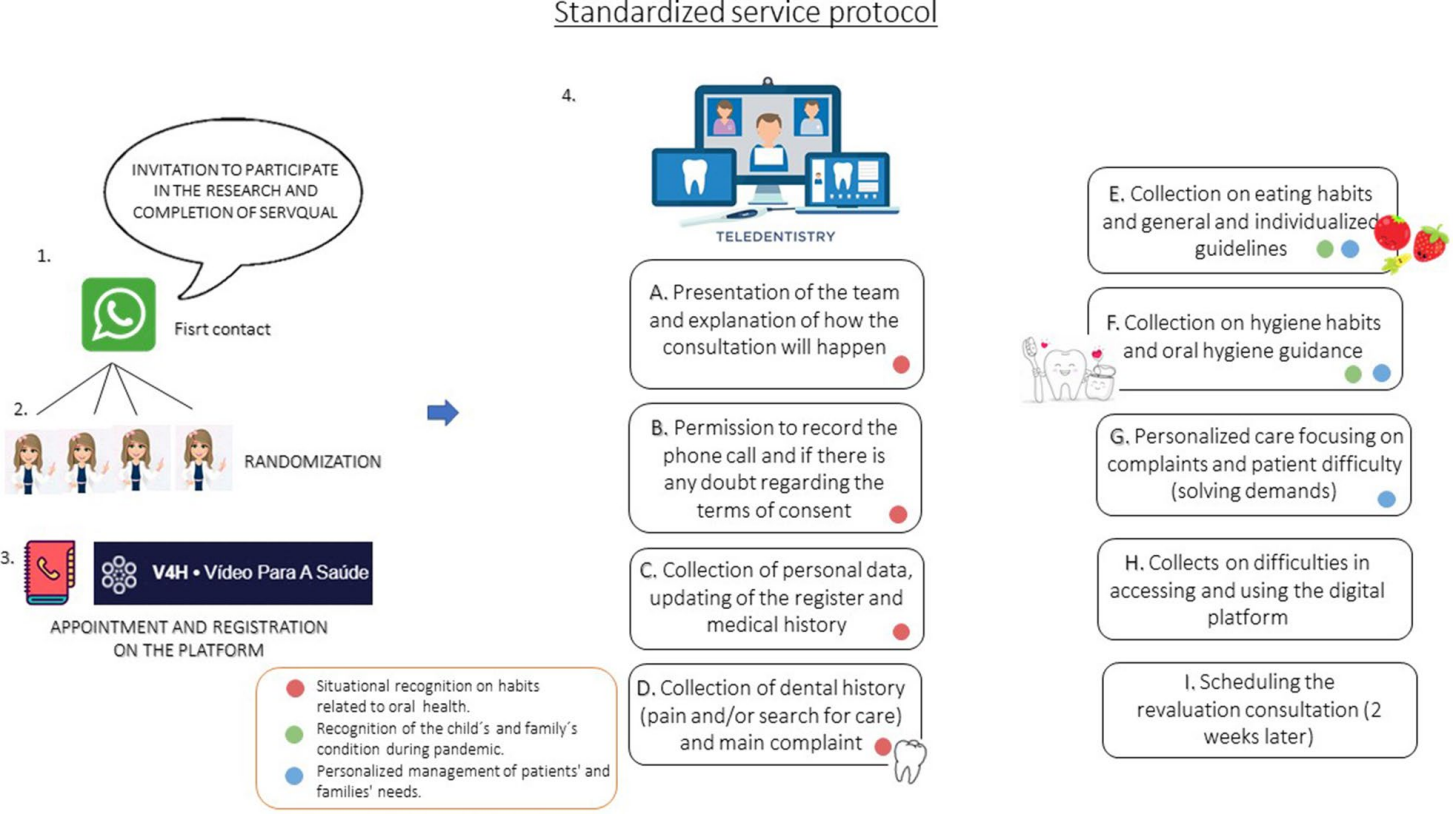
Standard Operational Procedures (SOP) that were used to guide the non-face-to-face consultation in the Teletrailer program

**Fig. 5:**
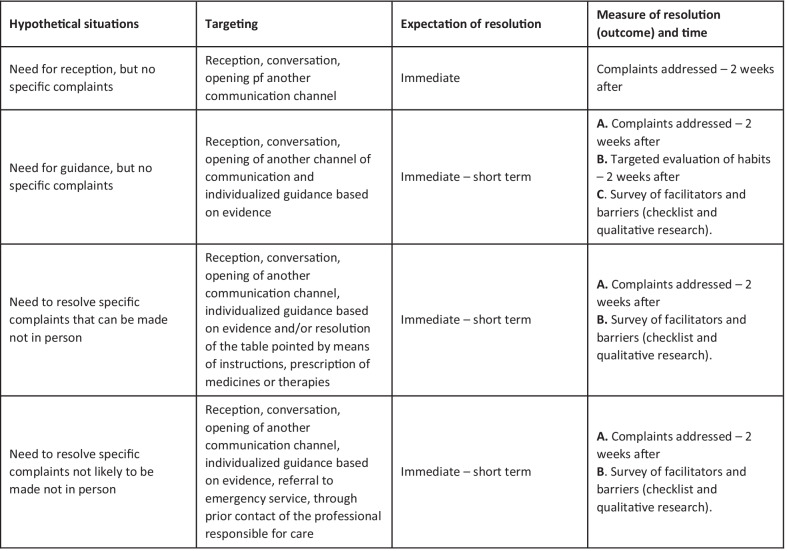
Clinical decision-making protocol to meet the patients’ and families’ reported demands related to oral care

The intervention is the non-face-to-face strategy used in the Teletrailer Program, based on teledentistry resources to minimize the interruption of primary dental care (prevention and curative) during the pandemic. The consultation was guided by a standardized protocol (Fig. 4) and performed by a team of dentists (graduate students), also responsible for previous face-to-face care at the unit. The session was divided into three parts (Fig. 4).

i. Recognition of the child’s and family’s condition during the pandemic (updates on medical history, current oral health condition, sought dental care in other units, need for referral, change in hygiene and diet habits).
ii. Situational recognition on habits related to oral health (diet, hygiene and other oral-related habits), followed by individualized orientations about them.
iii. Personalized management of patients’ and families’ needs according to their reports and complaints, looking to solve their specific demand. The management strategy will follow a pre-defined and standardized structure based on the best evidence available in the subject. Standard strategies should be used depending on which patients' report (Fig. 5).

An electronic form was created to guide consultation and permit data collection. The data collected by the form was stored under the supervision of the principal investigator (MMB), in cloud storage and on a USB stick.

The teleservice will be carried out through a digital platform (Video for Health—V4H). This platform is innovative, 100% Brazilian, created by the Research and Extension Center (Lavid) of the Informatics Center in Federal University of Paraiba (UFPB) in partnership with the Federal University of São Paulo (Unifesp) and financially supported by the National Network of Teaching and Research (RNP). The Teledentistry Center of Dental School, University of São Paulo contributed to adapting platform functionalities to the context of the SUS and the private sector in Brazil. The platform offers confidentiality in data traffic between participants, preservation for the time it is necessary to recover recorded videos, secure recording (with encryption) and registration in blockchain or trust protocol and a distributed registration technology to guarantee security in using it.

The operators were trained to use the V4H platform resources by expert researchers in teleservice and V4H development and support staff. Besides, they will receive the necessary technical support from the V4H team during the study. They are also calibrated on following Standard Operational Procedures to guide the consultation in a standardized way. Families enrolled in the Teletrailer program will receive information and support to use the platform from the Teletrailer team.

#### Outcomes

As the primary outcome for this before-and-after study, we consider problem-solving, considering the participant as the measurement unit. We will consider all the complaints pointed out by patients and their families during the baseline consultation. To measure that, a 2-weeks-later interview using the same platform (V4H) will address aspects relevant to understanding if the patients' and families' demands are solved during this time. A checklist to identify possible barriers and facilitators related to each type of complaint will be employed on these interviews. The time to resolution of the complaint will be computed as a secondary outcome.

Children will also be asked to toothbrush and floss during this assessment consultation. An external assessor will check the problem-solving related to the demand for oral hygiene orientations. The guidelines for orientation will be used as the reference standard to check if the demand was solved. If the child fulfils all oriented aspects, the demand is fully solved. If at least one assessment criterion is fulfilled, it is considered a partially solved demand. Cases of non-compliance with intervention sessions or those families that give up the consultations were also considered to understand the studied service's implementation.

Other outcomes will be assessed as secondary ones. The teleservice platform will measure consultation time. Technological and non-technological difficulties during the consultation (absences or delays, non-compliance, difficulties in using the platform, accessing the internet or even the computer or mobile phone) will be registered by an external assessor in a specific file at the end of the online session. The caregivers' and child's perceptions on the use of technologies in primary dental care will be collected by specific questionnaire [21] in the two-week-later assessment.

The users' attitudes during the consultation will be evaluated. An assessor, who participates as a spectator in the videocall, will use a specific scale, PANAS [22] The scale records necessary actions to classify this outcome. Besides, a semi-structured interview using guided questions will also be performed. It intends to identify participants' and parents' perceptions and potential barriers and facilitators for receiving the non-face-to-face dental during the pandemic. Afterwards, an external examiner will assess the video recordings using the same scale and perform qualitative analysis. This external examiner will be not included in the consultation and is unaware of previous evaluations.

For this qualitative phase, individual interviews will be recorded and transcribed. For this, individual interviews will be recorded and transcribed. The software Maxqda® will be used for categorizing the recorded speeches and, later, a content analysis (CA) will be performed [23]. For this analysis, categories are created following the common themes that emerge from the transcribed text. The analysis technique consists of three major steps: (1) pre-analysis; (2) the exploitation of the material; (3) the treatment of results and interpretation. Thus, we hope to identify qualitative differences between the groups that received technology-mediated care during the pandemic period or not, which cannot be captured through quantitative methods.

Finally, long-term outcomes will also be collected. Increment in caries experience since the interruption of dental and increment (or changes) in the needs for dental treatment will be assessed one year after the intervention. Presential clinical examination will be performed focused using dmft and DMFT and dental treatment need (including untreated caries, need for restoration repair, need of gingival or periodontal treatment, need for extractions for any reason) at this time point. An examiner unaware of baseline conditions will assess these indices and needs. These outcomes will be compared to those previously recorded in participants' records, resulting in the difference between the indices between these moments.

#### Sample size and recruitment

As we have a demand study, all children enrolled for dental treatment at the dental unit will be potentially recruited for this before-and-after study and no sample size is required. The child will be our unit of analysis for that. As previously described, refusals and non-compliance with non-face-to-face consultations will also be registered as outcomes. At least five attempts to contact participants who accept to participate will be used. In these attempts, we will consider different manners of communication, including phone calls, text and Whatsapp messages, email messages (when possible).

### Randomized controlled trial (controlled by waiting list)

#### Main purpose

We outlined a randomized patient-controlled study on the waiting list for care to assess the impact of teleservice implementation under the patients' and their families' perspectives, permitting comparison with a reference, in this case, those patients who are not participating in the Teletrailer program yet.

#### Participants: eligibility criteria

Children eligible for the previous study are also eligible for this one and is moved forward to the randomization step. Similarly, those who cannot be contacted after several attempts will be excluded from the sample. Children or guardians who do not agree to participate in the research were excluded from this randomizable sample.

#### Interventions

The family (including all children enrolled at the unit from the same family) will be randomized using stratified randomization by one researcher to one of the groups (intervention vs waiting list). The stratification will be performed considering the dentist will provide the non-face-to-face consultation and the week for inclusion. In this way, balanced randomization was obtained per professional each week.

Randomization order will be generated by the website sealedenvelope.com. The list will be stored under the supervision of the person responsible for randomization (TGOM) and the principal investigator (MMB), in cloud storage and on a USB stick. The dentist who will provide the dental care will be unaware of randomization. They will receive the list with families/patients they are supposed to care for each week sent via email by the person in charge of randomization.

The two groups will differ according to the time of application of the intervention and outcome assessment. The non-face-to-face intervention group will receive the intervention immediately after randomization (t0). Then, after two weeks, the outcome will be measured as described further (t2). The waiting list group (control) will not receive the intervention at this time  and then assessed after the same period to permit controlling the answers (t2). Once this is done, the children on the waiting list will receive the intervention regularly (t3) and become part of the previously detailed list of before-and-after study participants. However, for the present RCT, only data without intervention will be considered (t2). The intervention will be provided as described in the before-and-after study *Interventions* section. Both groups will have been contacted by a research team member to explain the study and will have answered a baseline questionnaire previous to randomization (t-2). At this time, all participants will know the teleservice will be available, but they will not know when that will happen.

#### Outcomes

We set the family's perception of dental care quality as the  primary outcome. We will consider the caregiver's perspective (those who participate in the non-face-to-face consultation) measured two weeks after the intervention (teleservice test group) or two weeks after waiting for care (control group) (Figs. 2, 3). The SERVQUAL questionnaire will be used [24] (Additional file 3). SERVQUAL is a summary instrument of multiple scales, with a high level of reliability and validity already used in Portuguese [24]. It can be used to understand users' expectations and perceptions concerning a wide range of services. In the present study, it is supposed the participants evaluate the degree to which they perceived the proposed service.

The questionnaire consists of 22 items, divided into 5 dimensions related to the service (tangible aspects—appearance of physical facilities, equipment, personnel and communication materials; reliability—ability to perform the promised service safely and correctly; responsiveness—willingness to help clients and provide prompt service; assurance—knowledge and courtesy of employees and their ability to inspire trust and empathy—personalized attention given to customers) [25]. A total of 100 points is allocated among the five dimensions.

As secondary outcomes, we define the gap between users' perception and expectations, each partial assessment for five different domains included in the SERVQUAL questionnaire, and finally, the overall participants' perception of the service.

A model previously proposed was used to detect the gap between users' perceptions and expectations [26]. For that, the SERVQUAL questionnaire was applied in two different moments (before and after receiving care). Then, the difference between the scores that customers attribute to the different pairs of statements. After consent to participate in the study, the baseline questionnaire will be sent to participants when contacted to participate in the study. This baseline assessment (t-2) should be done two weeks before the allocation (t0). We will calculate the mean score attributed in the baseline SERVQUAL assessment (t2) in each dimension to evaluate individual dimensions separately. Finally, one extra item was included besides the mentioned questionnaire (t2): "How do you feel about these service attributes that are already provided?". It was scored on a 5-point Likert-type scale of "very bad (1)" to "very good (5)." to identify overall participants' perceptions.

#### Sample size and recruitment

The sample calculation was made using the application sample-size.net and considered the primary outcome for the RCT as a reference. As more than one dimension is considered in evaluating the primary outcome, we consider an average between them. A hypothetical user's difference between the perceived and the expected users' quality appraisal related to a regular health service clinic [27] was used to estimate a minimum difference for this trial, aiming at a more conservative calculation. The minimum difference expected was set, then, as 0,17 (effect size) and a standard deviation of 0,53 assumed (based on the worst case scenario in the referred study) [27]. A minimum sample of 306 users or family representatives in the RCT was estimated. Extra 20% was added to compensate for possible losses, resulting in a final sample of 368 families recruited. Thus, the first 368 families from the unit's care list will be included (randomized). To reach the required number of children, all participants registered at the mobile dental care unit will be invited to participate in this study by telephone or social media contact or message, as detailed in the before-and-after study.

#### Independent variables collection

For both clinical trials described, available data will be collected from a data repository (dental records) administered by the researchers. Variables as time since the last consultation, oral health conditions associated (previous needs, caries experience, treatment adhesion/retention, oral-related habits, socioeconomic factors) may be used as independent variables. In addition, other independent variables will be collected parallel to intervention (teleconsultation), as changes in habits during the pandemic, level of education, age and occupation of the caregiver users involved in teleservice and previous experiences in health assisted by technologies.

#### Statistical methods

We will adopt the intention to treat analysis for both clinical trials, and all recruited participants will be considered when expressing our findings. Imputations of the outcomes will be considered whenever missing data is detected. In these cases, using conditional imputation methods will be used.

##### Before-and-after study

Firstly, we will calculate the problem-solving rate achieved with non-face-to-face care. Besides, a subgroup analysis will consider different demands identified. We will also calculate the average time of resolution of the demands (difference between the moment of the appearance of the complaint or demand and the moment of its resolution) and the reduction of the waiting time (time-lapse from when the care is provided and the regular face-to-face activities re-opening).

Regarding children's and caregivers' perception, we will calculate the usability rate (ease of use reported by caregiver users), satisfaction with the tools, behavior towards proposed strategies, and difficulties encountered, dividing them into difficulties with technologies and not related to technologies. Regression models will demonstrate the association of demand solving and the independent variables mentioned above. The same statistical approach will be used to outcomes related to the children's and caregivers' perception (usability, satisfaction, and behaviour to the technologies with the resolution of cases) and the time to resolution of complaints will be considered a dependent variable of conditional risk models. The events will be attention needs and the time counted from the last face-to-face contact. In these models, we will explore as independent variables the other variables also mentioned above, as well as aspects related to the difficulties reported by users.

The qualitative content analysis of the non-face-to-face consultations mediated by technology will be descriptive. It will look for possible explanations to different caregivers' perceptions and identify barriers and facilitators associated with the type of care offered.

##### The RCT (waiting list controlled)

This study will first compare the satisfaction with the health care received using the SERVQUAL score between the groups (with and without technology-mediated care). We will use an appropriate statistical test depending on the distribution of scores. The scores assigned for the perception of each dimension will also be compared. Appropriate regression analyses may also be performed to verify other variables' influence, besides the group (test vs control) on users' perception of the received care.

We will also compare the gaps (difference between expected and perceived) between groups. Comparisons between the perceived and the expected in each one of the groups will also be performed. Once again, distributions will be checked to choose the appropriate statistical approach to be used.

##### Simulation study: prospecting the findings for the reality of the national health system

After collecting the effects of implementing the technology-assisted primary care strategy, we will simulate a situation adapted to the scenario of interest,  the national public health system, the SUS (Fig. 2). This strategy has been used to provide subsidies (measurements) related to changes safely and efficiently before they are implemented on a large scale [28].

e will use simulations considering the characteristics of the basic health units that provide primary dental care for children. We will create a specific model considering the distribution of variables in the sample for outcomes collected in trials but prospecting for a wider population eligible for primary care in SUS. We will use some index variables, e.g. caries experience, that we have collected in the trials and a similar one can also be found in official national data. The relationship between such variables and the studied outcomes will be investigated in our sample and will guide the model building.

Finally, using a bootstrap technique, we will simulate data equivalent to SUS reality, considering appropriate variables as reference for that, as mentioned. We will collect updated official data on the impact of the pandemic on the Brazilian public health system and data on oral health conditions in the age group obtained through the last oral health survey (SB Brasil 2010) [29]. We will assume the essential characteristics of these units, which will also be considered in this simulation model. Some scenarios will be set to represent different contexts from different Brazilian regions.

From this simulation study, we hope to find the mean values and their 95% confidence intervals related to the problem-solving rate, reduction of waiting for time and user satisfaction related to the implementation of technology-mediated health care in the primary care of children assisted by the SUS.

##### Economic and social impact assessment

Some economic evaluations were outlined to answer the efficiency of resource allocation when implementing teleassistance activities to solve the pent-up demand caused by the pandemic. To comprehend a broader overview of the economic impact of this implementation process, we will consider the different effects explored in the trials, both child- and parent-centred.

**Fig. 6:**
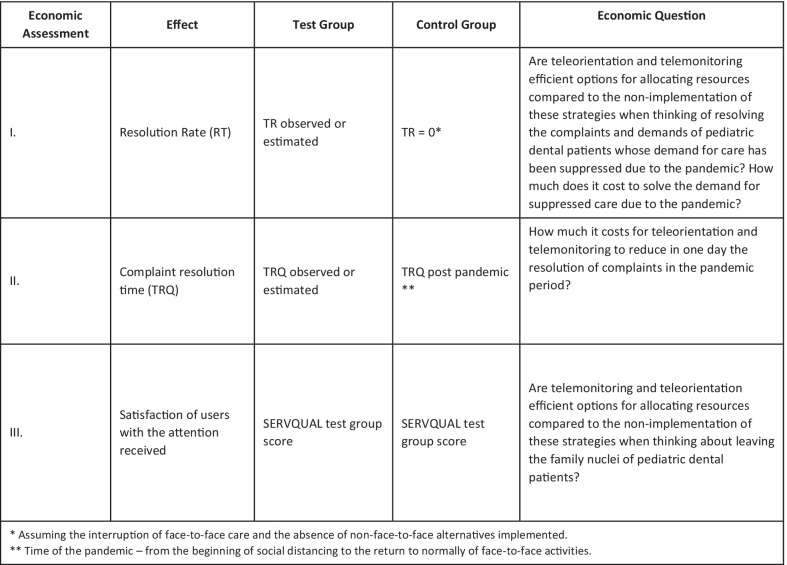
Economic evaluations (cost-effectiveness type), respective effects, questions to be answered and possible subgroup analyses foreseen

**Fig. 7:**
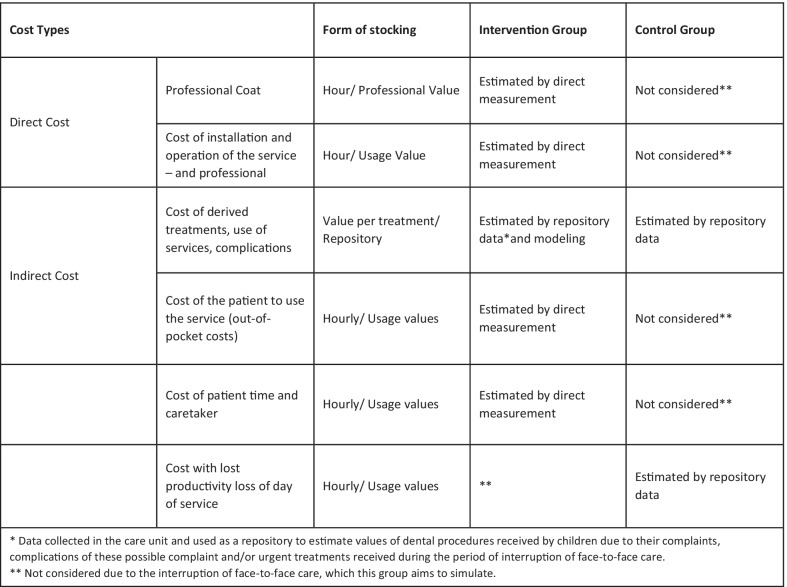
Cost components to be valued for the economic evaluations

(a) Perspective and Time Horizon: We will adopt a societal perspective for the analyses. The time horizon will be set as the trans-pandemic time, being limited by the beginning of social distancing in Brazil and the date of return to normality of activities officially determined for it according to official government recommendations, as end of the period of interest. Eventually, subgroup analyses will be considered for different Brazilian regions with different profiles regarding the duration of the pandemic. Alternatively, we may also model data using another time horizon, in which we consider the achievement of an ideal vaccination rate in Brazil (or in specific regions).

(b) Type of Evaluation/Effects: Three different trial-based economic evaluations will then be performed using cost-effectiveness approaches. A different effect will be used for each one (Fig. 6). Mainly, data will be extracted from trials described elsewhere in this protocol. Modelling strategies will be used to simulate more prolonged effects than those measured in the trials.

(c) Costs: The costs will be considered as described below for each assessment. The unit of analysis will be the research participant (patient/child) to which primary care was being directed and the strategy of micro-costing. Resources used will be valued as described in the Fig. 7. The direct costs will be those related to the provision and installation of services. Indirect costs will consider the patient's cost for the use of dental services or costs derived from care) that must be computed in a broader character (Fig. 5). All costs will be valued in Brazilian Real and converted into dollars.

Direct costs will be computed considering the time of use of the teleservice. This time will be recorded on the platform and by the service professional to double-check. For the calculation for the professional cost: the salaries of professionals will be based on the official national data will be used. With this, it will be multiplied by the time spent. Sensitivity analyses will be performed using data from different regions and/or different available services/positions.

The costs for the installation and maintenance of the teledentistry system take into account the program's implementation and its continuous operation. This type of cost will consider the time spent by the team for developing the system and the cost of maintaining and using the platform. An hourly value for using the technology will be calculated based on system capacity and usage flow data per month [30] and thus the value of the time obtained. A similar strategy will be made considering the expenses related to training and training of the team to implement the strategy [31].

To value the cost of using the technology by the patient/family, the average prices for 1Mbps of internet and Kwh of energy in Brazil, taking into account the time spent in each consultation. The value for patient's and caregiver's time and caregiver will be valued using arbitrary reference values (for example, the minimum wage and/or average salary of the population) and the time spent during care.

To value the treatments received outside the dental unit, the average value per procedure was obtained from a data repository of costs related to care provided at the same unit when performing another clinical study (NCT02473107). If a child needs to perform a restoration during the period, a value is estimated based on that repository.

In the case of unresolved complaints or patients who miss the expected follow-up until the end of the pandemic, the costs of possible complications will be valued based on the expected outcomes using models.

In the case of patients who will not receive the intervention, the costs of the intervention will not be considered since face-to-face care at the time of the pandemic is suspended (Fig. 5).

(e) Analysis Plan: The cost-effectiveness strategy will be used for economic analyses. Incremental values will be calculated both for costs and effects. Therefore, the difference between the new strategy and the one you want to replace will be calculated. (ΔE = E_with_tele_ – E_without_tele_ and ΔC = C_with_tele_ – C_without_tele._ The confidence intervals will be estimated, for each parameter, using the bootstrap technique and considering the sample values referring to costs, effects, incremental costs and incremental cost ratio incremental effectiveness.

**Fig. 8:**
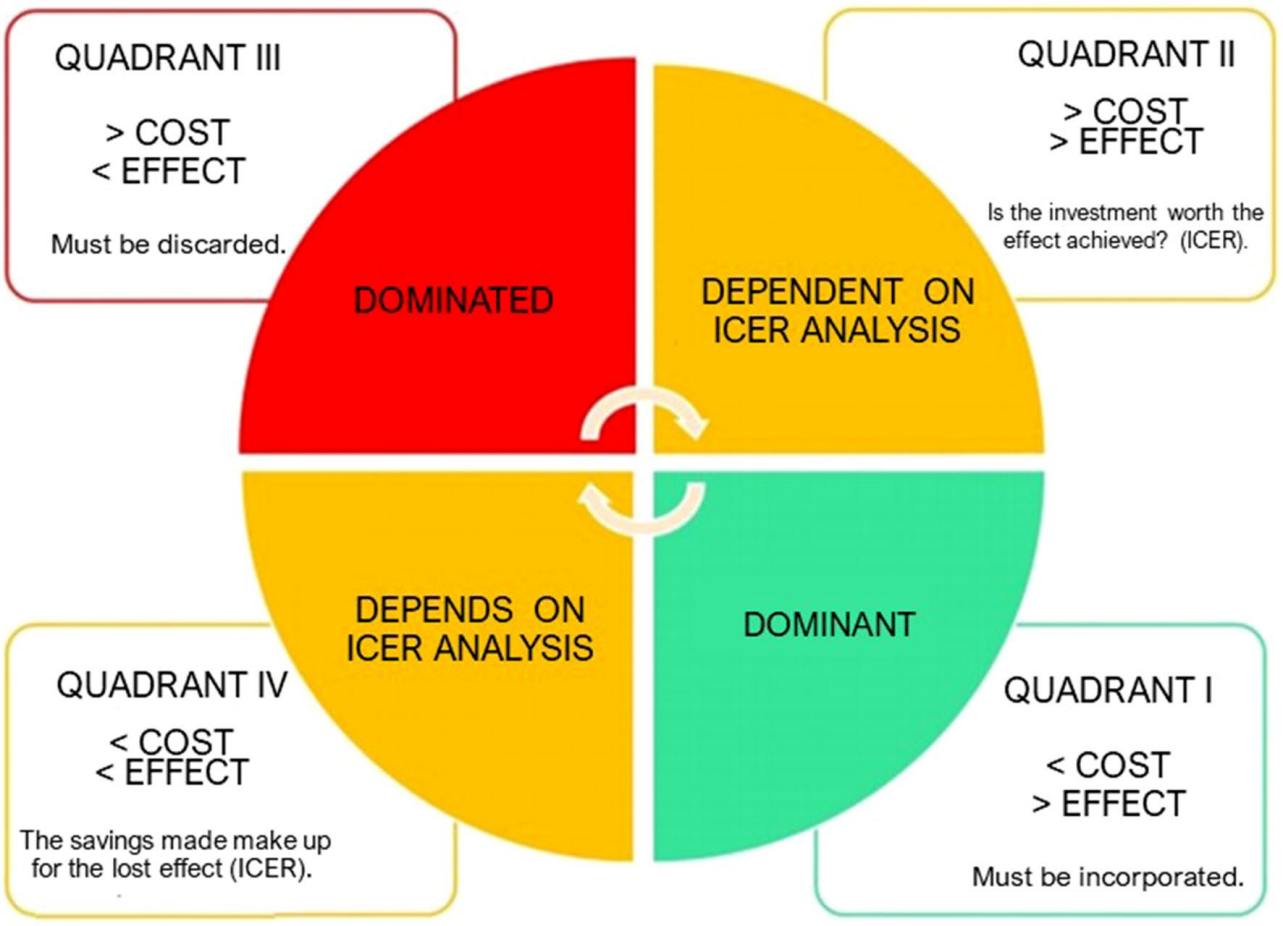
Diagram for interpretation of costs and effects in the cost-effectiveness plan

The decision-making will depend on the interpretation that will be made of the values collected in the cost-effectiveness plane. Depending on the quadrant in which these simulation results are placed, it may be called dominant or dominated, so an incremental-effectiveness cost ratio calculation may be required for the decision to be made. If the results are in the Northeast or Southwest quadrant (Fig. 8), the incremental cost-effectiveness ratio will help to decide whether or not the intervention will be cost-effective, that is, whether the extra expenditure attributed to the strategy is really worth the effect achieved by it, or even, whether what will be saved with the new strategy is worth the loss that has in its effect.

Acceptability curves will be plotted considering different ceiling ratios. This strategy will be used for each tested effect to check the strategy's cost-effectiveness in different economic evaluations.

(f) Sensitivity analyses: The economic evaluations will initially be evaluated considering a SUS-simulated scenario in which non-face-to-face care is implemented. The confidence limits of the parameters used (effects and costs) will be considered for performing deterministic sensitivity analyses.

We will adopt a Bayesian strategy to probabilistic sensitivity analyses to explore the uncertainties of health effects and costs [32–34]. For this, following the sample distribution, we will create simulations for both effects and costs, using XLSTAT 2021 (Addinsoft, Paris, France) and the effects and incremental costs. Also, the distributions of these parameters will be considered when the probabilistic analyses are performed.

Next, we will also perform other sensitivity analyses, considering the values prospected for the national health system, considering its magnitude and peculiarities. If necessary, subgroup analyses may be adopted to address specific Brazilian regions.

Finally, we will also analyze the expected value of perfect information (EVPI) and budget impact analysis (BIA). The first analysis (EVPI) allows us to know the value attributed to lousy decision-making due to the uncertainties presented in the data when the strategy is incorporated. It also allows us to estimate the value of the information (VOI), allowing the manager to have tangible arguments to decide whether there is interest in searching for evidence for the subject [35]. On the other hand, the BIA is an elemental analysis for planning and dimensioning the expenses inherent to incorporating a new strategy in the health system [36].

Still considering the above scenarios (dental reference unit and SUS), we will use the economic modelling strategy to consider some aspects not directly measurable in the evaluations described above but relevant from the point of view of decision-making. For this purpose, in the model, we will consider the costs and effects (including risks and benefits) inherent to the face-to-face resolution of some complaints and demands that could be resolved in the clinical studies described above.

To construct and validate these models, a group of experts, including experts in economic assessments, in evidence-based practice, service representatives and managers/decision-makers will be involved. The values related to the probability of occurrence of the different effects considered, as well as the costs derived from each of the situations or states included in the decision tree, will be obtained from the clinical studies conducted in the format of face-to-face care and also official data sources or pertinent bibliography. If necessary, data compilation will be done in meta-analysis format for a more robust estimate. Once this is done, the same analytical strategies previously scored will be employed.

Suppose a potential positive effect of the technology applied to primary dental care for children is detected. Then, models to understand how these tools could be used in the post-pandemic period to optimize the care and demand of this type of care within the SUS. For this, new decision trees will be proposed considering for each type of relevant demand (reception, guidance, resolution of some complaints without face-to-face need), possibilities of evolution, leading to inherent effects and costs. Then, another economic evaluation considering the economic impact of maintaining these technological tools within the context of primary care in the SUS in the post-pandemic period may be carried out. Such models may consider values related to the family's need for going to a dental unit for being cared for, caregivers' productivity losses because they accompany children in some visits, increase of dentist's productivity, possible losses due to difficulties in access and technological domain, among other possible points raised in clinical studies and also by the experts involved in the construction of the theoretical framework and validation of the models.

For the proposed frameworks, Markov models will be used, constructed and rotated in the appropriate software TreeAge. Annual cycles will be established and, a priori, a 10-year time horizon will be considered. The base case and health states will also be defined, given what was observed in previous studies. In these models, we will try to contemplate quantitative aspects (size of the population of children in the age group covered by the system) and qualitative (regional specificities) characteristics of the SUS. Exploratory analyses will be made considering possible variations in time horizons.

To verify if the insertion of technologies in primary dental care will impact social inequalities, we will analyze how some parameters associated with access may influence studied outcomes. Participants will be divided into groups considering socioeconomic factors, such as income, maternal education. Further, we will test if there are differences, for example, regarding the difficulties of access to technologies for care and guidance in primary care between these groups. We will collect pertinent data from the clinical studies and consider the mathematically simulated data for prospecting the reality to the national health system.

We will use managed entry agreement schemes to deal with the risk of heterogeneity of findings in economic analyses [37] adapted to the Brazilian reality. At this stage, we will consider the models and decision tress built to check the efficiency of resource allocation when implementing technologies in the management of relevant situations in the face of the COVID-19 pandemic and possible subsequent implementation in the SUS. The risks of uncertainty will be quantified using an instrument proposed for this purpose [38], and its actual impact can be evidenced for the health system managers, for example. That will also be a way to compare the different strategies in different environments (settings) to be incorporated within the national public health system.

## Discussion

Given the scenario of COVID-19, in which social distancing has been necessary, the professionals had to review their conduct to minimize the chances of contagion with the virus. Thus, Teledentistry has been gaining prominence, as it is an innovative method of providing health services that allow contact guidance without the need for face-to-face care [39].

On the other hand, because it is an innovative type of care, many issues are being raised around the telehealth. While teleservice support is argued to improve equitable access to health care, it may also cause disparities in access due to digital difficulty [40, 41]. It is a fact that this type of care presents some challenges, not only related to digital complications, access, connection, but also oncerning the performance of a preliminary physical examination, difficulty in visualization, difficulty in diagnosis. But even so, telehealth can contribute substantially to finding a balance between face-to-face encounters and remote follow-up of patients, especially in pandemic moments [42]. These findings demonstrate the need for evidences who actually support the use of such strategies in primary dental care.

In Brazil, we have not found many studies related to the implementation of Teledentistry, especially with children. This series of studies on the implementation of non-face-to-face care may bring essential contributions to understanding how such technologic inclusion in dental practice could result. For that, both different study designs and different outcomes are being explored in this protocol. As we designed here, small-scale studies can more closely approximate the clinical or community context of an RCT and may be helpful to test some aspects of intervention feasibility [14]. Looking at "how does strategy may work" for performing care for children when a face-to-face consultation is not feasible is our big deal in this protocol. As "how it works" may be answered in different ways [14], we opted for exploring aspects that matter to the dentist and the patient/family and society in general.

For deciding to include this type of technology (or not) in the health system, or even in any dental practice, we need information related to acceptability, demand, costs, implementation power, efficacy. All these areas may be addressed for feasibility studies [14]. It is expected that with the results of this project, we can contribute scientifically to all these issues, and permanently modify the work process of oral health teams in Brazil, and maybe, in other parts of the world.

The teleservice is not a new tool in health care. However, the COVID-19 pandemic created an opportunity window that makes this approach more visible and probably desirable. We believe after this pandemic and returning of regular clinical appointments, some positive lessons may become perennial. Thus, it is also crucial to understand how all these efforts performed during this atypical period may contribute to the real-life after the pandemic ends.

Another important mission in this protocol is bringing out robust findings that may induce policies and show experiences serve as examples even for other countries. We reinforce the responsibility and mission to generate knowledge with public resources and disseminate them at scale so that good practices can be carried out to ensure benefits to all SUS users, prospecting and expanding contributions, possibly, to different realities related to the SUS within Brazil or even, throughout the world.

## Supplementary Information

**Additional file 1.** SPIRIT checklist.

**Additional file 2.** Checklist for Health Economics Analysis Plans (HEAPs) for trial-based economic evaluations [20].

**Additional file 3.** SERVQUAL questionnaire (English version).

## Data Availability

The datasets used and/or analyzed during the current study are available from the corresponding author on reasonable request.

## Abbreviations

BIA: Budget impact analysis
CA: Content analysis
CI: Informatics center
EVPI: Expect value of perfect information
FAPESP: São Paulo State Research Foundation
FOUSP: University of São Paulo School of Dentistry
LAVID: Research and extensions center
ICTS: Information and communication technologies
RNP: National network of teaching and research
SPIRIT: Standard protocol items: recommendations for interventional trials
SUS: Unified health system
V4H: Video for health
VOI: Value of the information
