# Supplementary material for: Using information and communication technologies (ICTs) to solve the repressed demand for primary dental care in the Brazilian Unified Health System due to the COVID-19 pandemic: a randomized controlled study protocol nested with a before-and-after study including economic analysis
Source: BMC Oral Health. 2022 Apr 7;22:112. doi: 10.1186/s12903-022-02101-9 (PMC8988474; doi:10.1186/s12903-022-02101-9)
Supplement: Supplementary file 2 — Additional file 2. Checklist for Health Economics Analysis Plans (HEAPs) for trial-based economic evaluations [20]. [file 12903_2022_2101_MOESM2_ESM.docx]

**Supplementary material 1. Health Economics Analysis Plan (HEAP) template v1.0**

The full list of essential and optional items is given below, with expanded item descriptions and practical examples of how the item might appear in a HEAP. Please note that the examples are drawn from a number of different studies.^†^

**Essential items**

|  |  | **Description** | **Example** |
| --- | --- | --- | --- |
| **Section 1: Administrative information** | | | |
| 1.1 | Title | Title that matches protocol and which includes the phrase ‘Health Economics Analysis Plan' | Using information and communication technologies (ICTs) to solve the repressed demand for primary dental care in the Brazilian Unified Health System due to the COVID-19 pandemic: a randomized controlled study protocol nested with before-and-after study including economic analysis. |
| 1.2 | Trial registration number | Trial registration number and name of registry that uniquely identifies the clinical trial on a publicly accessible registry (and other relevant trial study numbers) | Registration NCT04798599 |
| 1.3 | Source of funding | Name of funders for trial and economic evaluation and funder(s)’ reference number(s) | FAPESP-2012/50716-0 and Coordination for the Improvement of Higher Education Personnel (CAPES) (2017-2022). |
| 1.4 | Purpose of HEAP | Brief statement of the purpose of the HEAP | The present trial aims to elucidate the impact of implementing the ICTs in primary dental care for children on resolving the pent-up demand for primary dental care to children in the SUS due to the COVID-19 pandemic. |
| 1.5 | Trial protocol version | Trial protocol version number associated with this HEAP | This document has been written based on information contained in the trial protocol version 1, dated 23 February 2022. |
| 1.6 | Trial Statistical Analysis Plan (SAP) version | SAP version number associated with this HEAP | SAP Version: 1.0, Date: 23 February 2022 |
| 1.7 | Trial HEAP version | Sequential number and date of this version | HEAP Version: 1.0, Date: 23 February 2022. |
| 1.8 | HEAP revisions | Date, justification for revision and summary of changes to the HEAP. Specify the individual making any revisions/changes to the HEAP. | *The HEAP revision history can be presented in tabular format with the following column headings:*  Updated HEAP Version No; Protocol version; Section number changed; Description of, and reason for, change; Individual making the change (e.g. Dr [name] made the revision before any comparative analyses had been conducted); Date changed.  Each row subsequently added to the table will indicate each HEAP revision change. |
| 1.9 | Roles and responsibilities | Names, affiliations and roles of individuals who have significantly contributed to the HEAP | This study protocol was prepared by Karina Haibara De Natal and approved by, Prof/Dr Mariana Minatel Braga. The trial health economist(s) Mariana Minatel Braga are responsible for conducting and reporting the economic evaluation in accordance with the HEAP. |
| 1.10a | Signature(s) of person(s) writing HEAP | Signature(s) of the person(s) writing the HEAP (and date) | 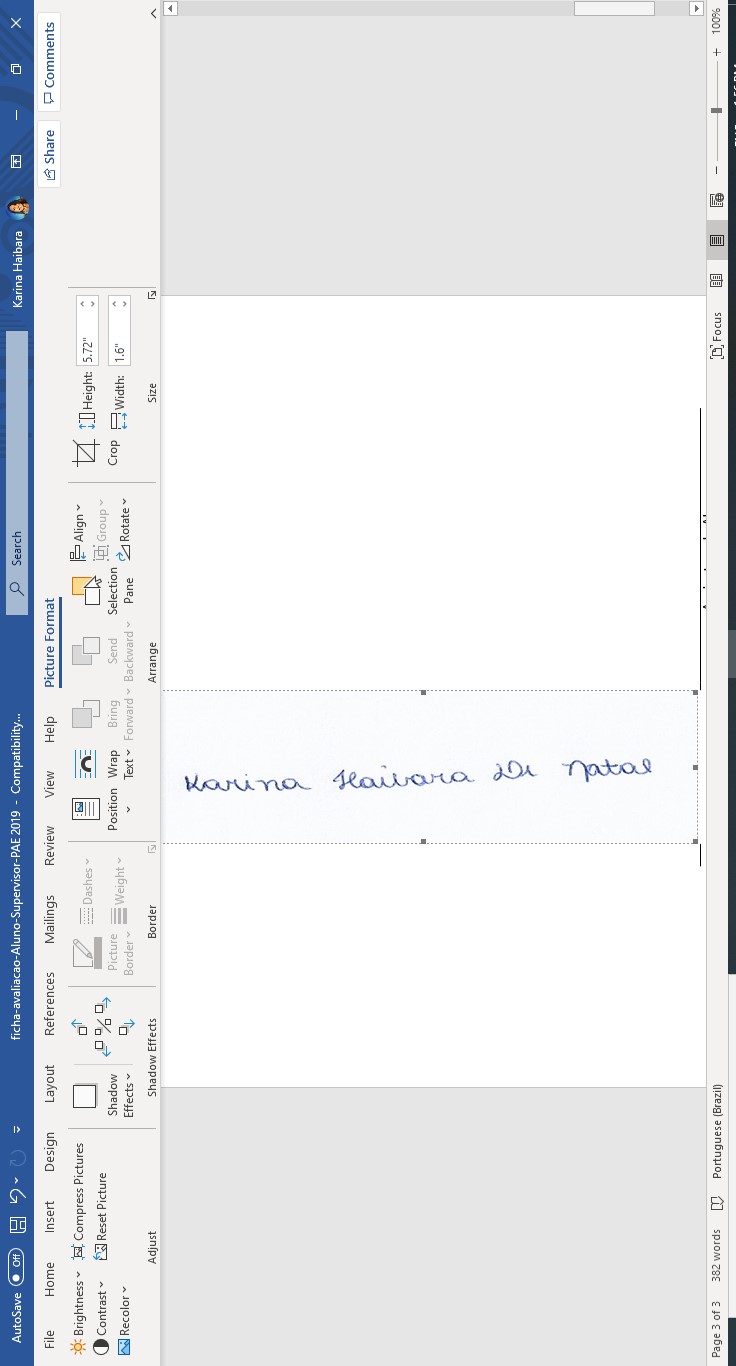  23/02/2022    23/02/2022 |
| 1.10b | Signature of senior health economist | Signature of senior health economist who is guarantor of the economic evaluation (and date) |   23/02/2022 |
| 1.10c | Signature of Chief Investigator | Signature of the Chief Investigator for the trial (and date) |   23/02/2022 |
| **Section 2: Trial introduction & background** | | | |
| 2.1 | Trial background and rationale | Synopsis of trial background and rationale including a brief description of research question and brief justification for undertaking the trial | With the COVID-19 pandemic, thousands of children had their dental care interrupted or postponed, generating a pent-up demand for primary care. To minimize the impact of this interruption of face-to-face care, information and communication technologies can be an alternative and even likely to be envisioned within the Unified Health System (SUS). In this sense, this study sought to show the impact of the use of information and communication technologies (ICT) in the resolution of the repressed demand for primary dental care to children in the SUS, due to the COVID-19 pandemic proposing the use of telemonitoring, teleorientation and telescreening to solve meet the demands arising from this interruption in primary elective care. |
| 2.2 | Aim(s) of the trial | Clearly and briefly state the main aim(s) of the trial | This proposal has a general objective to show the impact of the use of information and communication technologies (ICT) on the resolution of the pent-up demand for primary dental care for children in the SUS, due to the COVID-19 pandemic. |
| 2.3 | Objectives and/or research hypotheses of the trial | Describe specific trial objectives (primary and secondary) or trial hypotheses | 1. To identify the repressed demand for the interruption of dental care and the benefits and difficulties, as well the perception of users, in the implementation of new strategies of non-face-to-face care based on technology (teleservice), using a cellular unit of primary care for the child and prospect, through models, this situation for the scenario of the Unified Health System. 2. Perform different forms of economic evaluation to measure whether the gains achieved with the implementation of the teleservice compensate for the additional costs possibly associated with it, or if the teleservice modalities are associated with the long-term resource savings, considering the scaled implementation for the Brazilian public health system. 3. Explore how the introduction of these technologies could benefit the Unified Health System in the trans and post-pandemic period, contribute to the correction of possible inequities in health care and other social aspects, as well as result in possible differences when exploring the different Brazilian scenarios.   . |
| 2.4 | Trial population | Describe the trial inclusion and exclusion criteria | All children enrolled for dental care in the unit were eligible for this trial. Their parents/caregivers will be contacted and invited to be part of the non-face-to-face program. After several attempts, children who cannot be contacted will be excluded from the sample. Children or guardians who do not agree to participate in the research were be computed as an outcome, as described further. In this case, teleconsultation may be performed if they desire, but without other data collection for research purposes. |
| 2.5 | Intervention(s) and comparator(s) | Describe the intervention(s) and comparator(s) | Intervention: The teleservice will be carried out through a digital platform (Video for Health -V4H). The service will consist of 3 parts: a) recognition of the condition identified in the pandemic period, b) realization of diet guidance, hygiene and other necessary habits, c) targeting the needs presented. The guidelines, although individualized for each child´s needs, will follow a pre-defined and standardized structure and based on the best evidence available on the subject. In the control group, children waiting for the intervention (under implementation in the unit due to the pandemic) will be evaluated for outcomes. |
| 2.6 | Trial design | Briefly describe the trial design including type of trial such as cluster, crossover, etc. Can also include details of power calculation, sample size (including any separate calculations for economic endpoints), randomisation and blinding. | Randomized, patient-controlled waiting list study nested with a before-after study to be conducted in a cellular unit, simulating a basic dental health unit. Next, a modeling study was conceived, prospecting the situation found for the reality and full demand of the Unified Health System. Expected sample of 368 families. Triple masking (participant, service professional and result evaluator). Simples randomization. |
| 2.7 | Trial start and end dates | Trial recruitment start and end dates and the follow-up period | Recruitment started in December 2020 and is due to finish in June 2021. The follow-up period will run for 12 months until December 2021. |
| **Section 3: Economic approach/overview** | | | |
| 3.1 | Aim(s) of economic evaluation | Describe the aim(s) of the economic evaluation | Are teleorientation and telemonitoring efficient options for allocating resources compared to the non-implementation of these strategies when thinking of resolving the complaints and demands of pediatric dental patients whose demand for care has been suppressed due to the pandemic? How much does it cost to solve the demand for suppressed care due to the pandemic? How much it costs for teleorientation and telemonitoring to reduce in one day the resolution of complaints in the pandemic period? Are telemonitoring and teleorientation efficient options for allocating resources compared to the non-implementation of these strategies when thinking about leaving the family nuclei of pediatric dental patients? |
| 3.2 | Objective(s) of economic evaluation | Describe the objectives (primary and secondary) of the economic evaluation | Perform different forms of economic evaluation to measure whether the gains achieved with the implementation of teleservice compensate for the additional costs possibly associated with it, or whether they are associated with a long-term resource savings, considering the implementation sized for the Brazilian public health system. Explore how the introduction of these technologies could benefit the SUS in the trans and post-pandemic period, contribute to the correction of possible inequities in the health care and other social aspects, as well as result in possible differences when different Brazilian scenarios are explored. |
| 3.3 | Overview of economic analysis | Briefly outline and justify the type of economic evaluation to be undertaken, identifying the primary economic analysis and outlining the analysis plan and the methods that will be used | Three economic evaluations will then be performed, using cost-effectiveness analyses. These assessments will be based on a clinical study and will include, for prospecting for longer effects, associated economic modelling strategies. The unit of analysis will be the research participant (patient) to which primary care was being directed and the strategy of micro-costing. |
| 3.4 | Jurisdiction(s) | Specify the jurisdiction(s) in which the analysis will be conducted including details of the country(s) and health system(s) | The trial is conducted in Brazil which has one the largest public health systems (SUS). |
| 3.5 | Perspective(s) | State the perspective(s) from which the economic analysis is being conducted, such as societal perspective and/or healthcare payer perspective | We will consider as perspective the society. |
| 3.6 | Time horizon(s) | State the time horizon(s) over which costs and consequences are being evaluated | As a time horizon, the trans-pandemic time, considering as limit the beginning of social distancing in the state and, as an end, the date of return to normality of activities officially determined for it, according to the São Paulo Plan (green phase). |
| **Section 4: Economic data collection & management** | | | |
| 4.1 | Statistical software | Specify the statistical software that will be used to carry out the health economic analysis | XLSTAT 2021 (Addinsoft, Paris, France) |
| 4.2 | Identification of resources | Justify and describe items of resource use that will be measured as part of the trial | Provision and installation of services considering the time of use of the teleservice: professional cost, cost of installation and operation of the service.  Patient´s cost for the use of dental service: cost of derived treatments, use of services, complications; cost of the patient to the service; cost of patient time and caretaker; cost with lost productivity loss of day of service. |
| 4.3 | Measurement of resource-use data | Describe the resource-use data collection method(s) (including external routine datasets) and the time points at which they will be used. | Resource-use data will be collected util 12 months post randomisation and to value the treatments received outside the dental unit, the average value per procedure was obtained from a data repository of cost related to care provided at the same unit when performing another clinical study (NCT02473107). |
| 4.4 | Valuation of resource-use data | For each resource item measured, describe how the unit cost will be derived and from which specific price year. Outline how adjustments will be made for sources from different price years and which inflation index will be used. |  |
| 4.5 | Identification of outcome(s) | Specify and justify the outcome(s) that will be measured | Resolution Rate (How much does it cost to solve the demand for supressed care due to the pandemic?)Ç Complaint Resolution Time (How much it costs for teleorientation and telemonitoring to reduce in one day the resolution of complaints in the pandemic period?); Satisfaction of users with the attention received (Are teleorientation and telemonitoring efficient options for allocating resources compared to the non-implementation of these strategies?). |
| 4.6 | Measurement of outcome(s) | Describe the outcome data collection method(s) and the time points at which they will be used | The data will be collected during a year in which the teleservices will be made, a data repository held in the same unit-cell will be used to value calls offered in the non-face-to-face form. And they will be used after 12 months of study to perform the analyses. |
| 4.7 | Valuation of outcome(s) | For each outcome measured, describe how it will be valued and the source of these valuations | The cost-effectiveness strategy will be used for economic analyses. Incremental values will be calculated both for costs and effects. Therefore, the difference between the new strategy and the one you want to replace will be calculated. The confidence intervals will be estimated, for each parameter, using the bootstrap technique and considering the sample values referring to costs, effects, incremental costs, and incremental cost ratio incremental effectiveness. |
| **Section 5: Economic data analysis** | | | |
| 5.1 | Analysis population | Outline the analysis population that will be used in the economic base-case analysis (such as intention to treat, per protocol) | The full analysis set will include all randomised participants, which is in accordance with the “intention to treat” (ITT) principle. |
| 5.2 | Timing of analyses | Describe the timing of all planned analyses (e.g. interim and final analyses) | The time horizon will be set as the trans-pandemic time, considering as limits the beginning of social distancing in Brazil and as an end, the date of return to normality of activities officially determined for it according to official government recommendations. Alternatively, we may also model data using another time horizon, in which we consider the achievement of an ideal vaccination rate in Brazil (or in specific regions). The primary analyses will be performed after one year of study and data collection and the other analyses when the study is finalized according to the time horizon described above. |
| 5.3 | Discount rates for costs and benefits | Detail the source of, and justification for, discount rates used for costs and benefits |  |
| 5.4 | Cost-effectiveness threshold(s) | Detail the cost-effectiveness threshold(s) to be used in analysis/interpretation |  |
| 5.5 | Statistical decision rule(s) | Describe how inference will be drawn (e.g. significance level, confidence intervals or mean net benefit) | . |
| 5.6 | Analysis of resource use | Describe how differences in the use of resources/services between randomised groups will be compared |  |
| 5.7 | Analysis of costs | Describe analyses of the cost data, specifying any covariates for statistical adjustment, assumptions, and alternative methods |  |
| 5.8 | Analysis of outcomes | For each outcome used in the economic analysis, describe how the outcome will be analysed, specifying any covariates for statistical adjustment, assumptions, and alternative methods |  |
| 5.9 | Data cleaning for analysis | Outline how data will be cleaned before analysis |  |
| 5.10 | Missing data | Specify the procedure for dealing with missing data |  |
| 5.11 | Analysis of cost-effectiveness | Describe the methods that will be used to summarise cost-effectiveness. |  |
| 5.12 | Sampling uncertainty | Describe how uncertainty around the costs and effectiveness estimates and summary cost-effectiveness measures will be explored |  |
| 5.13 | Subgroup analyses or analysis of heterogeneity | Describe any analyses of subgroups or heterogeneity in cost-effectiveness and the analysis methods used | Subgroup analyses will be considered for different Brazilian regions with different profiles regarding the duration of the pandemic. |
| 5.14 | Sensitivity analyses | Describe any sensitivity analyses and their form | The economic evaluations will initially be evaluated considering a SUS-simulated scenario in which non-face-to-face care is implemented. The confidence limits of the parameters used (effects and costs) will be considered for performing deterministic sensitivity analyses.  We will adopt a Bayesian strategy to probabilistic sensitivity analyses to explore the uncertainties of health effects and costs. we will also perform other sensitivity analyses, considering the values prospected for the national health system, considering its magnitude and peculiarities. Finally, we will also analyse the expected value of perfect information (EVPI) and budget impact analysis (BIA). |
| **Section 6: Modelling** | | | |
| 6.1 | Extrapolation or decision analytic modelling | Outline whether decision analytic modelling or any other extrapolation will be used to estimate cost-effectiveness results beyond the period of the trial or to introduce an additional comparator or other evidence. | We will simulate a situation adapted to the scenario of interest (the national public health system), the SUS. We will use simulations considering the characteristics of the basic health units that provide primary dental care for children. We also using a bootstrapping technique, we will simulate data equivalent to SUS reality |
| 6.2 | Model type | Describe the modelling approach that will be used and duration of extrapolation | We will create a specific model considering the distribution of variables in the sample for outcomes collected in trials but prospecting for a wider population eligible for primary care in SUS. We will use some index variables, e.g. caries experience, that we have collected in the trials and can also be found in official national data. |
| 6.3 | Model structure | Detail the model structure (where possible, include diagram of model states and transitions between them) |  |
| 6.4 | Treatment effect beyond the end of the trial | Describe the duration and size of treatment effect in the period beyond the end of the trial |  |
| 6.5 | Other key assumptions | List the key structural assumptions of the model |  |
| 6.6 | Methods for identifying and estimating parameters | For each model parameter, describe the methods and data sources that will be used to estimate the parameter (e.g. from the RCT, systematic review, meta-analysis, other published data or expert opinion) |  |
| 6.7 | Model uncertainty | Describe the methods that will be used to assess parameter uncertainty in the results. Describe sensitivity analyses for the impact of other types of uncertainty on results. |  |
| 6.8 | Model validation | Describe the methods and data that will be used to check the face, internal and external validity of the model |  |
| 6.9 | Subgroup analyses/heterogeneity | Describe subgroup or heterogeneity analyses that will be executed and reported within the extrapolation or decision analytic modelling |  |
| **Section 7: Reporting/publishing** | | | |
| 7.1 | Reporting standards | Describe any guidelines that will be followed when publishing results | CHEERS guidelines will be followed when reporting the health economic evaluation, in a format appropriate to stakeholders and policy makers. |
| 7.2 | Deviations from the HEAP | Describe the procedure for reporting any deviations from the HEAP | Any deviation from HEAP will be described and justified in the final published report. |
| **Section 8: Appendices** | | | |
| 8.1 | Health economic collection tools | Include template examples of the resource-use data collection sheets and resource-use questionnaires | The data will be collected at the Mobile Dental Unit of FOUSP located in the municipality of Barueri. A dental record via Google Forms was made to guide the teleservice. SERVQUAL questionnaire was used to analyzed the perception of parents in relation to the service. |

**Optional items**

|  |  | **Description** | **Example** |
| --- | --- | --- | --- |
| **Section 1: Administrative information** | | | |
| O1.1 | Table of contents | List of HEAP contents with page numbers |  |
| O1.2 | Abbreviations/glossary of terms/definitions | List of abbreviations and/or acronyms used within the HEAP alongside their meanings/definitions | SUS: Unified Health System  EVPI: Expected Value of Perfect Information  BIA: Budget Impact Analysis  VOI: Value of the Information |
| **Section 4: Economic data collection & management** | | | |
| O4.1 | Monitoring collection of health economic data | Outline how the health economic data collected will be monitored | The data from this study will be collected by a protuary via Google Forms and the V4H (Video For Health) platform, which records all teleservice and times related to it. Platform development data will be collected by the V4H team. And the procedures performed in person, and other costs related to the cost of the professional/patient will be consulted in a database of the unit-cell of this study obtained with previous studies. |
| O4.2 | Database management | Outline how the economic data will be stored and managed and by whom | The data will be stored in clouds and USB device with access only from the main researcher of this study (MMB). |
| O4.3 | Data entry | Outline how data will be entered/handled and outline any checking systems in place |  |
| O4.4 | Data archiving | State whether datasets, interim datasets and final analysis will be archived, and if so, how |  |
| **Section 6: Modelling** | | | |
| O6.1 | Value of information analysis | Describe whether value of information analysis is planned and the type and methods that will be used to calculate value of information |  |
| **Section 8: Appendices** | | | |
| O8.1 | Cross-referencing to other trial documents | Reference to other relevant trial documents that are adhered to and followed when writing the HEAP and any other references used when writing the HEAP |  |
| O8.2 | Illustrations | Illustrations such as annotated questionnaires detailing the database fieldnames, flow charts outlining the flow of data for the economic evaluation, or template tables | Attached to paper. |

**†** Examples were extracted, and in some cases modified, from existing draft or final HEAPs provided to the study team. We are grateful to the trial teams from which they originated.
