# Supplementary material for: Using information and communication technologies (ICTs) to solve the repressed demand for primary dental care in the Brazilian Unified Health System due to the COVID-19 pandemic: a randomized controlled study protocol nested with a before-and-after study including economic analysis
Source: BMC Oral Health. 2022 Apr 7;22:112. doi: 10.1186/s12903-022-02101-9 (PMC8988474; doi:10.1186/s12903-022-02101-9)
Supplement: Supplementary file 3 — Additional file 3. SERVQUAL questionnaire (English version). [file 12903_2022_2101_MOESM3_ESM.docx]

ATTACHMENT 2 – SERVQUAL questionnaire applied in this protocol

QUESTIONÁRIO SERVQUAL APLICADO ANTES DO ATENDIMENTO

SERVQUAL QUESTIONNAIRE APPLIED **BEFORE** CARE

Patient name:

Name of the person responsible:

Degree of kinship: ( ) mother ( ) father ( ) grandparents ( ) cousin ( ) brother ( ) other

- What grade would you give for the care your child receives?

Being 1 the worst grade and 7 the best grade: ( ) 1 ( ) 2 ( ) 3 ( ) 4 ( ) 5 ( ) 6 ( ) 7

- What grade would you give to the facility, staff and materials offered for your child´s oral treatment?

Being 1 the worst grade and 7 the best grade: ( ) 1 ( ) 2 ( ) 3 ( ) 4 ( ) 5 ( ) 6 ( ) 7

- What grade would you give for the waiting time between one appointment and the other?

Being 1 the worst grade and 7 the best grade: ( ) 1 ( ) 2 ( ) 3 ( ) 4 ( ) 5 ( ) 6 ( ) 7

- What grade would you give for the duration of each query?

Being 1 the worst grade and 7 the best grade: ( ) 1 ( ) 2 ( ) 3 ( ) 4 ( ) 5 ( ) 6 ( ) 7

- What grade would you give for the behavior of dentists in the care?

Being 1 the worst grade and 7 the best grade: ( ) 1 ( ) 2 ( ) 3 ( ) 4 ( ) 5 ( ) 6 ( ) 7

- What note would you give to the team´s willingness in response and take your questions?

Being 1 the worst grade and 7 the best grade: ( ) 1 ( ) 2 ( ) 3 ( ) 4 ( ) 5 ( ) 6 ( ) 7

What grade would you give to the knowledge offered by the team and to answer your questions?

Being 1 the worst grade and 7 the best grade: ( ) 1 ( ) 2 ( ) 3 ( ) 4 ( ) 5 ( ) 6 ( ) 7

- What note would you give for the personalized attention offered to your child?

Being 1 the worst grade and 7 the best grade: ( ) 1 ( ) 2 ( ) 3 ( ) 4 ( ) 5 ( ) 6 ( ) 7

- What grade would you give for service hours?

Being 1 the worst grade and 7 the best grade: ( ) 1 ( ) 2 ( ) 3 ( ) 4 ( ) 5 ( ) 6 ( ) 7

- What grade would you give to supply your child´s needs?

Being 1 the worst grade and 7 the best grade: ( ) 1 ( ) 2 ( ) 3 ( ) 4 ( ) 5 ( ) 6 ( ) 7

SERVQUAL QUESTIONNAIRE APPLIED **AFTER** ATTENDANCE

Patient name:

Name of the person responsible:

Degree of kinship: ( ) mother ( ) father ( ) grandparents ( ) cousin ( ) brother ( ) other

- From 1 to 7, what note would you give for the waiting time between one query and the other?

( ) 1 ( ) 2 ( ) 3 ( ) 4 ( ) 5 ( ) 6 ( ) 7

- From 1 to 7, you would give that note for the personalized attention your child receives?

( ) 1 ( ) 2 ( ) 3 ( ) 4 ( ) 5 ( ) 6 ( ) 7

- From 1 to 7, on the supply of your child´s needs, what grade would you give?

( ) 1 ( ) 2 ( ) 3 ( ) 4 ( ) 5 ( ) 6 ( ) 7

**-** From 1 to 7, in relation to the duration of each query, what grade would you give?

( ) 1 ( ) 2 ( ) 3 ( ) 4 ( ) 5 ( ) 6 ( ) 7

- From 1 to 7, in relation to the behavior of dentists in the visits, what grade would you give?

( ) 1 ( ) 2 ( ) 3 ( ) 4 ( ) 5 ( ) 6 ( ) 7

- From 1 to 7, regarding the installation, the staff and materials offered for your child´s oral treatment, what grade would you give?

( ) 1 ( ) 2 ( ) 3 ( ) 4 ( ) 5 ( ) 6 ( ) 7

- De 1 a 7, em relação à disposição da equipe em responder e tirar suas dúvidas, qual nota você daria?

( ) 1 ( ) 2 ( ) 3 ( ) 4 ( ) 5 ( ) 6 ( ) 7

- De 1 a 7, em relação aos horários de atendimento, qual nota você daria?

( ) 1 ( ) 2 ( ) 3 ( ) 4 ( ) 5 ( ) 6 ( ) 7

- From 1 to 7, in relation to the knowlegde offered by the team to answer your questions, what grade would you give?

( ) 1 ( ) 2 ( ) 3 ( ) 4 ( ) 5 ( ) 6 ( ) 7
